# The sensitivity and specificity of using the McGill pain subscale for diagnosing neuropathic and non-neuropathic chronic pain in the total joint arthroplasty population

**DOI:** 10.1186/s40945-023-00164-7

**Published:** 2023-04-24

**Authors:** Dragana Boljanovic-Susic, Christina Ziebart, Joy MacDermid, Justin de Beer, Danielle Petruccelli, Linda J. Woodhouse

**Affiliations:** 1 Department of Rehabilitation, Sunnybrook Holland Orthopaedic & Arthritic Centre, Toronto, ON Canada; 2 School of Rehabilitation Sciences, McMaster University, Hamilton, ON Canada; 3 Department of Rehabilitation, Western University, 1151 Richmond St. London, ON N6A 3K7 London, Canada; 4 Hamilton Arthroplasty Group, Hamilton Health Sciences Juravinski Hospital, Hamilton, ON Canada; 5 Faculty of Rehabilitation Medicine, University of Alberta, Edmonton, AB Canada; 6 School of Physiotherapy and Exercise Science, Curtin University, Perth, Australia; 7 McCaig Institute for Bone and Joint Health Calgary, Calgary, AB Canada

**Keywords:** Pain, Arthroplasty, Sensitivity, Specificity

## Abstract

**Background:**

The purpose of this study was to describe the diagnostic performance of the Neuropathic Pain Subscale of McGill [NP-MPQ (SF-2)] and the Self-Administered Leeds Assessment of Neuropathic Symptoms and Signs (S-LANSS) questionnaire in differentiating people with neuropathic chronic pain post total joint arthroplasty (TJA).

**Methods:**

This study was a survey of a cohort of individuals who had undergone primary, unilateral total knee, or hip joint arthroplasty. The questionnaires were administered by mail. The time interval from operation to the completion of the postal survey varied from 1.5 to 3.5 years post-surgery. Receiver Operating Characteristic (ROC) analysis was used to assess the overall diagnostic power and determine the optimal threshold value of the NP-MPQ (SF-2) in identification of neuropathic pain.

**Results:**

S-LANSS identified 19 subjects (28%) as having neuropathic pain (NP), while NP-MPQ (SF-2) subscale identified 29 (43%). When using the S-LANSS as the reference standard, a Receiver Operating Characteristic (ROC) analysis for NP-MPQ (SF-2) had an area under the curve of 0.89 (95% CI: 0.82, 0.97); a cut off score of 0.91 NP-MPQ (SF-2) maximized sensitivity (89.5%) and specificity (75.0%). Correlation between the measures was moderate (r = 0.56; 95% CI: 0.40, 0.68).

**Conclusion:**

These finding suggest some conceptual overlap but some variability in diagnosis of NP which may relate to scale-tapping into different dimensions of the pain experience, or the different scoring metrics.

**Supplementary Information:**

The online version contains supplementary material available at 10.1186/s40945-023-00164-7.



## Introduction

Total joint arthroplasty (TJA) procedures are a safe and cost-effective treatment for those with end stage osteoarthritis (OA) [1, 2]. Effectiveness of the procedure, combined with an aging population will result in increased utilization of joint arthroplasty. In terms of total hip and the knee replacements, reports from Canadian Institute of Health Information (2019) indicate that there has been an 17% increase in these procedures in the last five years. The projected estimates for the year 2030 indicate that total hip arthroplasty (THA) procedures would increase by 174% and total knee arthroplasty (TKA) by 674% [3]. Despite the overall effectiveness of arthroplasty procedures, current reports suggest that a considerable number of patients (14–28%) continue to report persistent or chronic pain, even though their damaged joint has been replaced [4, 5]. Furthermore, a recent review implies that among patients with severe persistent post TJA pain, there might be a subset of those who present with neuropathic pain (NP) type characteristics [5]. It is important to identify people with NP since the treatment needs and outcomes are different than other types of pain.

Detection of NP is challenging, in part due to the lack of indication of causality [6] and good case identification instruments [7]. With no definite physiological indicators of NP, diagnosis relies on characterization of the pain as neuropathic [6, 8]. Literature indicates that the pain experience is multi-dimensional and its characterization extends beyond the pain intensity [6, 9]. However, despite the agreement that different types of pain are associated with various pain characteristics, the research on distinctive pain qualities appears to be limited [8, 10]. Differentiating between NP and non-NP in individuals post TJA is crucial, as management of patients with chronic pain that manifests with neuropathic features requires different treatment strategies from chronic pain, which is mechanical in nature. Consequently, there is an unmet need to provide questionnaires to objectively identify those who develop chronic neuropathic pain after TJA.

To assist with classification, screening tools, such as the Self-Administered Leeds.

Assessment of Neuropathic Signs and Symptoms (S–LANSS) scale, have been designed to differentiate between NP and non-NP [11]. Diagnostic tools are designed to differentiate, and a result typically use yes/no responses. The latest revised version of the McGill’s Short Form Pain Questionnaire includes a subscale for identification of NP [12]. The modifications were made with intent to develop a single, comprehensive pain assessment tool for characterization of various types of pain. As an evaluative measure designed to detect change over time the items are scored 0–10, to support responsiveness. It is possible that evaluative measures could be used for diagnosis, if accurate cut-points can be identified that differentiate people.

The latest reports indicate that a considerable number of patients with chronic pain experience a combination of nociceptive and neuropathic pain features [6, 13, 14]. Thus, clinicians require a simple and accurate screening tool to identify patients with potential neuropathic pain in their daily clinical practice [6, 8, 15] since NP requires specific different treatment approaches than chronic nociceptive pain [10, 15, 16].

The purpose of this study was to evaluate the ability of the NP Subscale of McGill of the Short Form Version 2 (NP-MPQ (SF-2)) questionnaire to identify NP in individuals with chronic pain post TJA.

Our main objective was to evaluate the discriminatory power and diagnostic accuracy of the NP-MPQ (SF-2) subscale in classification of NP in individuals with chronic pain post TJA by examining rates of classification of NP, and by comparing the NP-MPQ (SF-2) to the S-LANSS as the reference standard. In addition, the association between the raw scores from the two measures [S-LANSS and NP-MPQ (SF-2)] was described.

## Materials & methods

### Design

This study was a survey of a cohort of individuals who had undergone primary, unilateral total knee, or hip joint arthroplasty. The project received approval from the human research ethics boards from the institution and the University.

### Subjects and study procedure

The subjects for the study were selected from the total joint arthroplasty (TJA) registry database acquired between 2007 and 2009 as part of an ongoing study. This registry, which includes demographics and outcome measure scores (self-reported pain and function [(Oxford Pain Scores (OPS), Harris Hip Scores (HHS), and Knee Society Score (KSS)) was retrospectively reviewed to identify potential subjects with chronic postoperative pain. Individuals were deemed to have chronic pain if their reported overall pain score was severe at 6 months or 1-year post-surgery, and that pain was the same or worse than reported preoperatively. Only individuals eligible for the postal survey completed the S-LANSS and NP-MPQ (SF-2).

Individuals who met the following inclusion criteria were contacted: they had undergone primary unilateral THA or TKA at least 6 months previously, they reported their postoperative pain level to be 3 or higher, out of 5, on the Oxford Pain Questionnaire. At either 6 months or 1-year post surgery their self-reported level of pain was the same or worse than it was preoperatively. Individuals were excluded if they had undergone total joint revision surgery, bilateral or staged arthroplasty, tibial or femoral osteotomy. The time interval from operation to the completion of the postal survey varied from 1.5 to 3.5 years postsurgery.

Of the 1143 TJA recipients, 148 individuals met all the inclusion criteria. Eligible participants received a letter in the mail from the surgeon co-investigator (JdB), who is also the Director responsible for the TJA database, inviting them to participate in this study. All potential participants were mailed information about the study and an informed consent form. Only those individuals who returned a signed written informed consent form were included in the study. Participants each received copies of both the S-LANSS and the Short Form McGill Pain Questionnaire via mail-in survey.

To increase the mail-in survey response rate, reminder notices and replacement questionnaires were sent to the nonrespondents 2–3 weeks after the initial mailing [17]. A total of three reminders were sent to improve the response rate [17]. All returned S-LANSS questionnaires were scored. Patients were classified as having neuropathic pain if their S-LANSS score was ≥ 12, according to a method proposed by Bennett and colleagues (2005) [11]. The NP-MPQ (SF-2) was scored according to the method proposed by Melzack [12].

### Instruments (primary outcome measures)

The S-LANSS is a seven-item questionnaire: five symptom items and two examination items are used to assess the patient’s NP status [11, 18, 19]. This scale is intended as a self-administered instrument for case identification based on a cutoff score. Scores range from 0 to 24, where a score ≥ 12 is indicative of neuropathic pain [11, 18]. The literature indicates that scores above the optimum cutoff score (≥ 12) when S-LANSS is self-administered are considered “S-LANSS positive” and very suggestive of neuropathic pain [11, 15]. The sensitivity and specificity of the S-LANSS when administered (to individuals with various chronic and neuropathic pain conditions including postsurgical patients) by mail has been reported in the literature as 74% (95% CI: 65, 83) and 76% (95% CI: 68, 85) respectively, compared to the clinical exam [11, 18, 19]. A recently completed project by the same authors as this manuscript determined that a neuropathic (NP) subtype was identified in 28% (S-LANSS ≥ 12). Internal consistency with Cronbach’s alpha of 0.76 was reported when the questionnaire was independently completed [19].

The McGill Pain Questionnaire Short Form-2 [MPQ (SF-2)] is a tool designed to provide information about the overall intensity as well as the quality of pain (sensory and affective). It consists of 22 experiences and descriptors of pain (18 sensory and 4 affective). The pain scores are derived from the sum of the intensity rating on a 10-point intensity scale (0 represents no pain, 10 represents the worst possible pain). Both subscales and total scores are calculated by taking a mean of all the item ratings [12]. This tool is a revision of the original short form McGill Pain Questionnaire, which was modified by adding 7 questions related to neuropathic pain. In addition, the original 4-point rating scale was replaced with a 0–10-point numeric rating scale for each question [12]. Good cross-sectional construct validity of MPQ (SF-2) with the Brief Pain Inventory and Multidimensional Pain Inventory scales was reported in individuals with a variety of chronic pain syndromes [12]. The MPQ (SF-2) has been reported as able to discriminate between those with painful diabetic peripheral neuropathy vs. those with diverse chronic pain syndromes [12]. A recently completed project by the same authors as this manuscript determined that a neuropathic (NP) subtype was identified in 43% (NP-MPQ (SF-2) ≥ 0.91).

### Secondary Outcome measure

The Oxford Questionnaires are joint specific twelve-item numeric rating scales (1–5) developed for assessment of patient’s perception of pain and disability in those undergoing total hip [Oxford Hip Score (OHS)] or knee [Oxford Knee Score (OKS)] replacement [14, 20]. Scores range from 12 to 60 with a higher score representing a greater level of perceived disability. The first ten questions are the same for both scales, while the remaining two are specific to the hip or knee joint, respectively. Good internal consistency after surgery, with a range of reported Cronbach’s alpha between 0.84 and 0.93 in individuals 3 to 24 months post THA has been documented for the OHS [21]. In addition, research has documented that the OHS is highly sensitive to change in patients undergoing THA [22] and that scores have a high correlation (rs = 0.7, *p* < 0.001) with Harris Hip Scores [23]. Similarly, the Oxford Knee Score has been shown to have good test-retest reliability in groups and individuals post TKA [23]. Good responsiveness to change in patients 6–12 months post TKA has also been documented for OKS [24].

### Analysis

The collected data from the postal survey were analyzed using the Statistical Package for the Social Sciences (SPSS) software version 19 (IBM SPSS Inc., Chicago IL). Double data entries were performed by random inspection of the paper surveys against the database entry in SPSS (version 19, IBM). To summarize the demographics of the sample population, univariate descriptive statistics were performed for all the analyzed variables.

The survey response rate was calculated as the number of questionnaires mailed out (*n* = 148) minus the number returned with an incorrect address (*n* = 4), minus the number returned with a statement that the addressee was unable to complete it because of death or incapacity (*n* = 9) and minus the ineligible participants (*n* = 6) [25].

S-LANSS scores were used to classify participants as having positive or negative findings for the presence of predominantly neuropathic (S-LANSS scores ≥ 12) or non-neuropathic (S-LANSS scores < 12) pain syndromes, based on recent reports that a score of 12 or greater when S-LANSS is self-administered are suggestive of neuropathic pain [11].

Receiver Operating Characteristic (ROC) analysis was used to assess the overall diagnostic power and determine the optimal threshold value of the NP-MPQ (SF-2) in identification of NP in individuals with chronic pain post total hip or knee arthroplasty (by using the S-LANSS scale as a “reference standard” for classification of NP). The optimal threshold value for NP-MPQ (SF-2) in classification of NP was established based on the visual assessment of the closest distance from the left upper corner (Area Under the Curve -AUC) and by examining the table for the curve coordinates. AUC is a method for evaluating the accuracy of a diagnostic test in differentiating between individual with and without the disease. The following guide has been suggested for categorizing the accuracy of a diagnostic test: AUC of 0.9–1.0 is an excellent test, 0.8–0.9 good, 0.7–0.8 fair, 0.6–0.7 poor while the 0.5–0.6 is considered a meaningless test [26].

Pearson Product Moment Correlation Coefficient was used to evaluate the association between two scales [S-LANSS and NP-MPQ (SF-2)]

Differences between the groups of TJA with NP vs. Non-NP were deemed to be significant at *p* < 0.05.

## Results

**Fig. 1:**
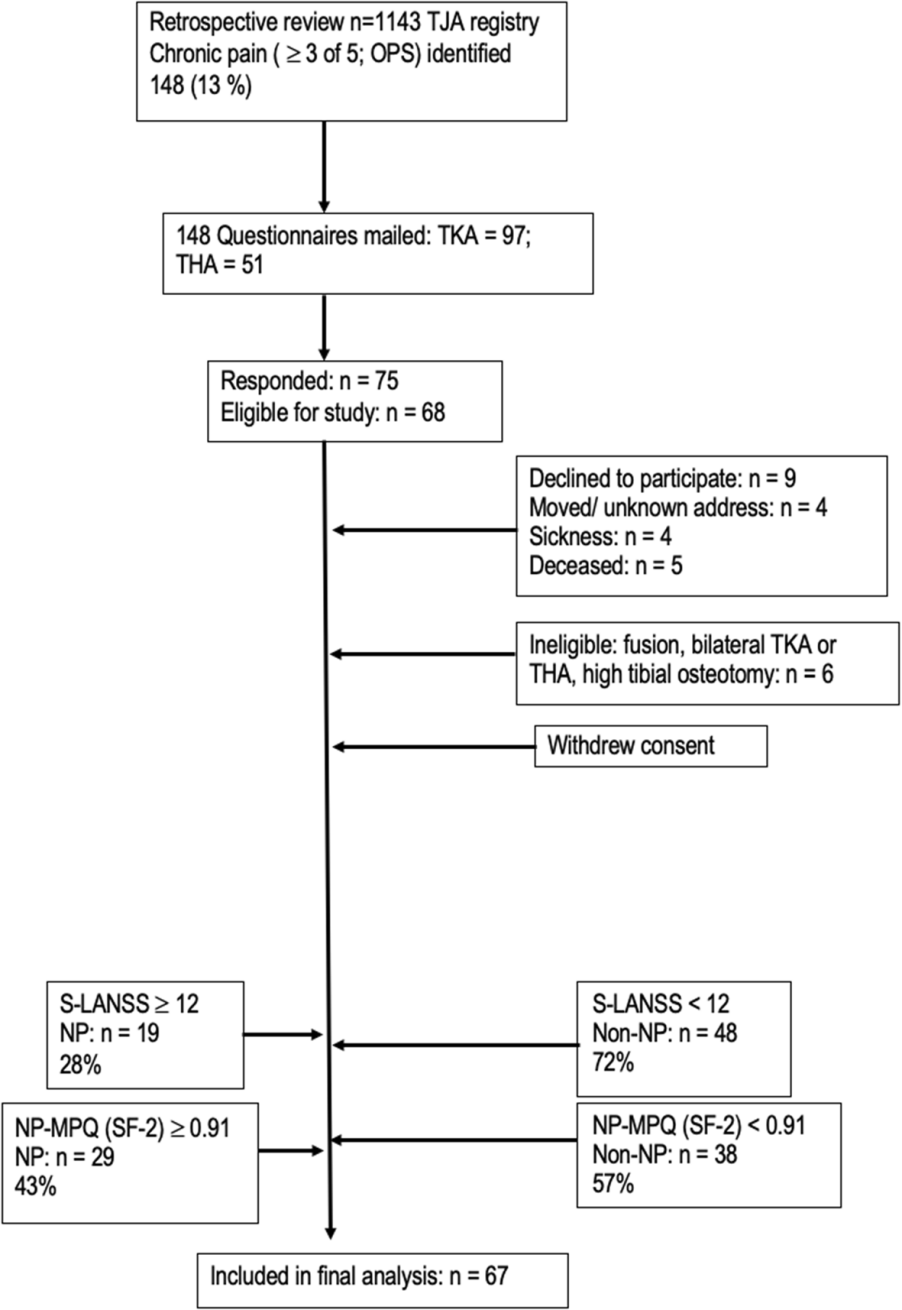
Study flow chart

Seventy-five patients completed the survey (58%) (Fig. 1, Flow chart summarizes the survey). Six responders were deemed ineligible (i.e., bilateral joint arthroplasty, surgeries, fusions, or osteotomies) and excluded from the analysis. One subject withdrew their consent after the completion of the survey. We had one individual who did not complete the S-LANSS questionnaire (missing). Thus, data from 67 subjects was included in the final analysis. Participant ages ranged from 37 to 88 years with a mean age of 70 (SD = 9.3) years, Table 1.

**Table 1:** Participant Demographics

TJA Individuals (*n* = 67)	TKA (*n* = 42) 63%	THA (*n* = 25) 37%
Count (percent)		
Males	15 (36%)	15 (60%)
Females	27 (64)	10 (40)
Mean (SD)		
Age (years)	69.67 (10.45)	70.28 (7.05)
BMI (kg/m^2^)	31.83 (6.38)	31.11 (5.74)
S-LANSS	10.19 (7.58)	4.80 (6.75)
NP-MPQ (SF-2)	1.73 (1.87)	0.81 (1.58)
Pre-Operative Oxford Scores	37.02 (8.05)	35.92 (9.40)

Abbreviations: *BMI *Body Mass Index; Oxford Scores [(12–60), higher score = greater disability]; *S-LANSS *Self-Assessment of Leads Neuropathic Signs & Symptoms [(0–24), scores ≥ 12 indicative of Neuropathic Pain (NP)]; NP Subscale of McGill’s Pain Questionnaire –Shot Form 2 (NP-MPQ (SF-2)) [(0–6), scores ≥ 0.91 indicative of NP].

### The presence of predominantly neuropathic (S-LANSS scores ≥ 12) or non-neuropathic (S-LANSS scores < 12) pain syndromes

Based on the S-LANSS, 19 subjects (28%) scored ≥ 12 and were classified as having neuropathic pain. When the NP-MPQ (SF-2) questionnaire was used, 29 subjects (43%) scored ≥ 0.91 and were classified as having NP. Mean (SD) score on the S-LANSS was 8.18 (7.69) and 1.39 (1.81) on the NP-MPQ (SF-2) subscale.

### Receiver operating characteristic (ROC) to assess the threshold value of the NP-MPQ (SF-2)

**Fig. 2:**
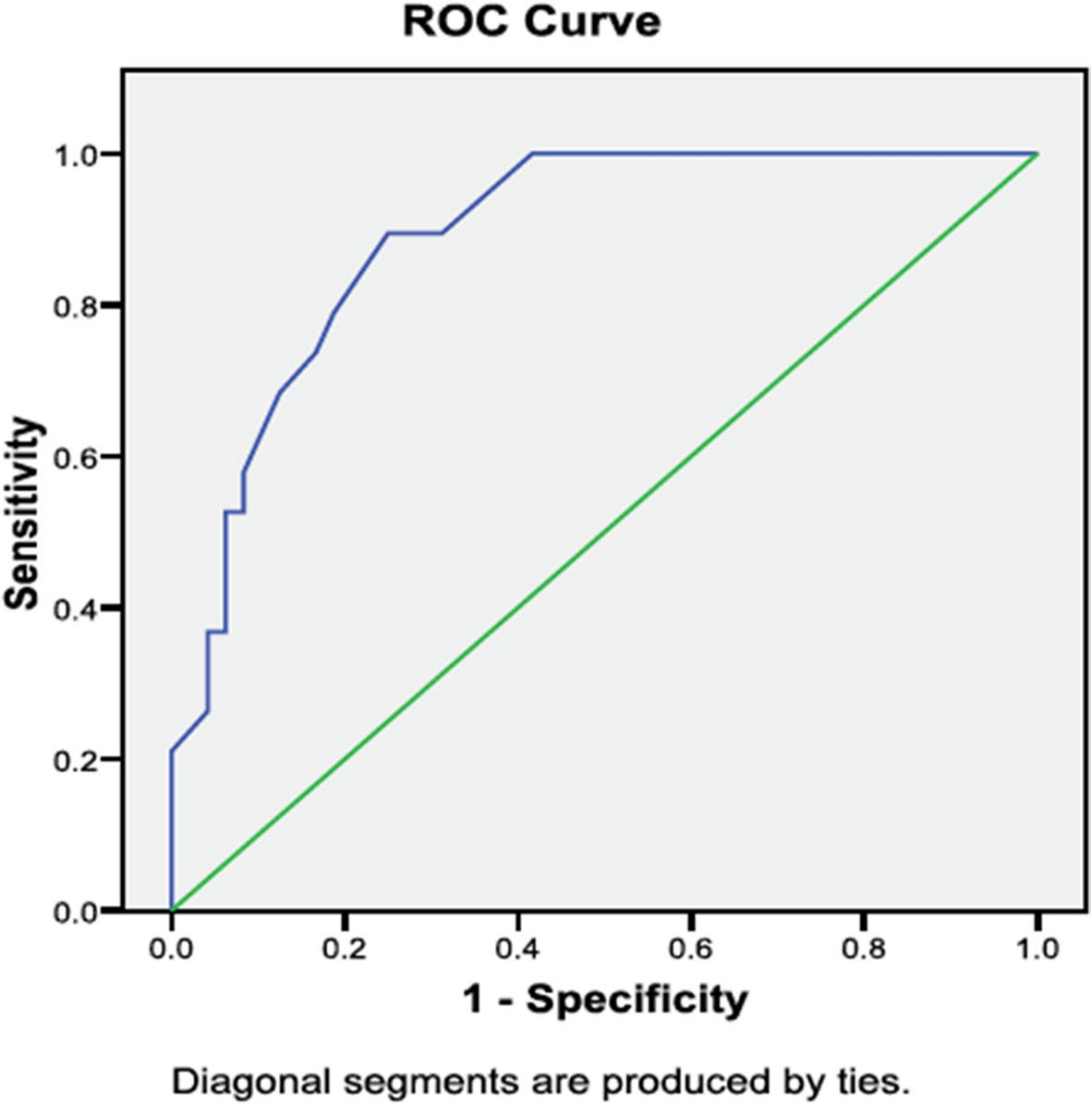
ROC indicating the ability of NP-MPQ (SF-2) subscale to discriminate between patients with NP and Non-NP pain 2 years post TJA

The Area under the curve (AUC) for the ROC curve comparing different cutoffs of the NP-MPQ (SF-2) yielded a good AUC = 0.89 (95% CI: 0.82, 0.97). The optimal NP-MPQ (SF-2) subscale cut-off score (Table 2) that maximized sensitivity (89.5%) and specificity (75.0%) was 0.91 points (ROC curve, Fig. 2).

**Table 2:** Example of different cut-off scores on the prediction of NP

Cut off Score	Sensitivity %	1- Specificity %
-1.00	100	100
0.80	100	46.0
0.24	100	50.0
0.41	100	55.0
0.58	100	59.0
0.74	89.5	68.7
0.91	89.5	75.0
1.08	78.9	81.2
1.24	73.7	83.3
1.41	68.4	87.5
1.58	63.2	89.6
2.66	52.6	17.0
2.99	52.6	37.0
3.49	36.8	37.0
4.83	26.3	58.0
5.88	10.5	0.00

### Association between NP-MPQ (SF-2) & S-LANSS scales

The Pearson Product Moment Correlation Coefficients were r = 0.56 (95% CI: 0.40, 0.68), indicating a moderate association between scores on the two measures.

## Discussion

This study found that the NP-MPQ (SF-2) subscale demonstrated good diagnostic accuracy (AUC = 0.89) in classification of NP vs. non-NP in patients with chronic pain patients post-total joint arthroplasty when the S-LANNS was regarded as the “reference standard”. In the absence of gold standard, an imperfect but accepted reference standard is typically used when validating newer measures [27]. Given the specific design of the S-LANNS for diagnosis and its previous validation [26, 27], it was chosen as the best available reference standard. When comparing two imperfect diagnostic measures, differences in diagnosis may reflect flaws in either tool. The fact the NP-MPQ (SF-2) classified more people with chronic post TJA pain as having the NP subtype suggests it has a bias in over -estimation or is more sensitive.

Screening tools such as S-LANSS have been recommended for identification of NP especially for clinicians [8]. However, literature indicates that in comparison to a clinician assessment, the S-LANSS and NP-MPQ screening tools miss detection of 20% of patients with NP features. Given that a clinician assessment has a level of subjectivity and that agreement between clinicians is imperfect, this is still an imperfect reference standard. It will be important to compare the NP-MPQ (SF-2) to an expert clinician diagnosis, which would help us understand the higher rates of diagnosis of NP with the NP-MPQ (SF-2) reflecting better detection or a biased misclassification error [28].

We chose a ROC analysis to evaluate the sensitivity and specificity of the NP-MPQ (SF-2) because it is considered a robust analysis, with the ability to evaluate accuracy across a range of different scores [29]. This method is commonly used to identify “cut-off score” or optimal threshold values for classification of those with a disease (true positives) vs. those without disease (true negatives) [29]. The optimal threshold value achieves a balance between sensitivity and specificity that allows optimal differentiation between those with and without the disease [29, 30]. The cut-off scores allowed us to dichotomize the 0–10 scores of the NP-MPQ (SF-2) into NP and non-NP groups. With our sample we were able to determine an optimal cut-off score (0.91) for NP-MPQ (SF-2) that maximized the balance between sensitivity (89.5%) and specificity (75.0%) in classification of NP vs. Non-NP in a TJA population. Based on these results, NP-MPQ (SF-2) classified more individuals with NP in comparison to the S-LANSS (reported 74% sensitivity and 76% specificity in postal survey). Thus, indicating that NP-MPQ (SF-2) would identify more individuals with NP in comparison to the S-LANSS scale.

Even though our primary interest in this study was the extent to which the NP-MPQ (SF-2) subscale classified patients in a similar way to the S-LANSS; the association between the two scales was explored. We found a moderate correlation between these two measures, which may reflect the difference in items and scoring. Moderate association between the tool scores supports a shared construct, but the differences in classification should be expected.

The findings of this study should consider some limitations. One of our main limitations is that there is no gold standard, and that we used an accepted but imperfect reference standard, the S-LANSS [11].

Although we started with a large pool of data (*n* = 1143), we focused on the smaller subset of patients who had worsening pain following arthroplasty (*n* = 148); and then conducted a follow-up survey that had a 53% response rate. A larger sample would have provided a more precise and stable ROC analysis. Finally, these data were acquired via mail in survey and were dependent on the accuracy of the responses provided, and we did not have a clinician examination as a reference standard.

## Conclusion and future recommendations

Despite TJA being the most common elective surgery in North America and Europe [2, 31] and evidence that persistent pain occurs in a subset of people to have TJA, we do not yet understand how to predict, identify, or manage this subset. Correct classification of individuals in a clinically feasible manner is an important step in early management [29]. Since the NP-MPQ (SF-2) can be used to assess people throughout the continuum of OA management, it could be an ongoing monitoring tool for the presence of NP in a clinically feasible manner. In this study and others, we often do not know if NP was present before surgery.

Our study indicated that there are a significant number of those who experience NP post TJA [as classified by S-LANSS and NP-MPQ (SF-2) scales respectively] in the group of chronic pain sufferers. Therefore, better tracking and early diagnosis is an important issue for people with OA.

Although this study focused on the classification of postoperative patients, a useful extension of this work would be to test the ability of the questionnaire to classify patients as having (or not) neuropathic features prior to surgery. In summary, our study suggests that the NP-MPQ (SF-2) may be useful in monitoring patients with OA and detecting the subgroup with NP.

## Supplementary Information

**Additional file 1.**

## Data Availability

The datasets used and/or analyzed during the current study are available from the corresponding author on reasonable request.

## Abbreviations

TJA: Total Joint Arthroplasty
OA: Osteoarthritis
THA: Total Hip Arthroplasty
TKA: Total Knee Arthroplasty
NP: Neuropathic Pain
S-LANSS: Self-administered Leeds Assessment of Neuropathic Signs and Symptoms
NP-MPQ (SF-2): Neuropathic Pain subscale of McGill Short Form version 2
OPS: Oxford Pain Score
HHS: Harris Hip Score
KSS: Knee Society Score
MPQ (SF-2): McGill Pain Questionnaire Short Form Version 2
OKS: Oxford Knee Score
OHS: Oxford Hip Score
ROC: Receiver operating characteristic
AUC: Area Under the Curve
SD: Standard Deviation
BMI: Body Mass Index
CI: Confidence Interval
SPSS: Statistical Package for the Social Sciences
